# When it is available, will we take it? Social media users’ perception of hypothetical COVID-19 vaccine in Nigeria

**DOI:** 10.11604/pamj.2021.38.230.27325

**Published:** 2021-03-02

**Authors:** Yusuff Adebayo Adebisi, Aishat Jumoke Alaran, Obasanjo Afolabi Bolarinwa, Wuraola Akande-Sholabi, Don Eliseo Lucero-Prisno

**Affiliations:** 1 Department of Clinical Pharmacy and Pharmacy Administration, Faculty of Pharmacy, University of Ibadan, Ibadan, Nigeria,; 2 Faculty of Pharmaceutical Sciences, University of Ilorin, Ilorin, Nigeria,; 3 Department of Public Health Medicine, School of Public Health and Nursing, University of KwaZulu-Natal, Durban, South Africa,; 4 Department of Global Health and Development, London School of Hygiene and Tropical Medicine, London, United Kingdom

**Keywords:** COVID-19, vaccine acceptance, COVID-19 vaccine, social media users, vaccine hesitancy, Nigeria

## Abstract

**Introduction:**

COVID-19 pandemic is a global public health threat facing mankind. There is no specific antiviral treatment for COVID-19, and many vaccine candidates are currently under clinical trials. This study aimed to understand the perception of social media users regarding a hypothetical COVID-19 vaccine in Nigeria.

**Methods:**

we conducted a cross-sectional survey among social media users in Nigeria in August 2020 using an online questionnaire. The questionnaire includes sections on the demographic characteristics of the respondents and their perception regarding a hypothetical COVID-19 vaccine. A total of 517 respondents completed and returned the informed consent along with the questionnaire electronically. Data were coded and abstracted into Microsoft Excel spreadsheet and loaded into the STATA 14 software for final analysis.

**Results:**

the results showed that more than half of the respondents were male 294 (56.9%). Most of the respondents 385 (74.5%) intend to take the COVID-19 vaccine when it becomes available. Among the 132 respondents that would not take the COVID-19 vaccine, the major reason for non-acceptance was unreliability of the clinical trials 49 (37.1%), followed by the belief that their immune system is sufficient to combat the virus 36 (27.3%). We found a significant association between the age of the respondents and the COVID-19 vaccine acceptance (P-value=0.00) as well as geographical location and COVID-19 vaccine acceptance (P-value=0.02).

**Conclusion:**

it was observed that most of the respondents were willing to take the COVID-19 vaccine. Our findings also reiterate the need to reassure the public the benefits an effective and safe COVID-19 vaccine can reap for public health. There is a need for national health authorities in Nigeria to ensure public trust is earned and all communities, including the marginalized populations, are properly engaged to ensure an optimal COVID-19 vaccine acceptance.

## Introduction

The novel coronavirus disease 2019 (COVID-19) caused by severe acute respiratory coronavirus 2 was first discovered in Wuhan animal market, Hubei Province, China in December 2019 [1]. Since its discovery, it has spread to more than 200 countries around the world and has been declared a pandemic by the World Health Organization [2]. COVID-19 has had unprecedented impacts on health and well-being of people all over the world, as well as the economy of many countries [3,4]. In Nigeria, the index case was reported on 27 February 2020, and more than 50,000 cases and over a thousand deaths have been documented since then [5]. As with past outbreaks, the novel nature of the current coronavirus outbreak implied that no definitive therapy existed for its treatment, instead, empirical therapies are being employed to manage the disease [1]. The rapid spread of the virus and continuous increase in number of cases alongside partial and/or total lockdown protocols in most countries necessitate the urgent development of accurate diagnostic methods, effective treatments, and vaccines for the disease [6]. The long-term solution to COVID-19, however, would most likely be a safe, globally implemented vaccination program with a broad range of clinical and socioeconomic benefits [7]. It is of point to note that vaccination is one of the greatest achievements of modern medicine and it is the greatest human intervention besides clean water and sanitation [8].

Major infections such as smallpox and rinderpest have been eradicated worldwide due to vaccines, while polio has almost been eradicated except for Afghanistan and Pakistan where it is still endemic [9-11]. The impact of vaccination on vaccine-preventable diseases cannot be overemphasized. The incidence and prevalence of diseases such as cervical cancer, hepatitis, yellow fever, tuberculosis, cholera, and tetanus, among others, have been severely reduced due to vaccine availability [12]. Vaccination plays important roles in disease eradication, control of mortality, morbidity and complications, mitigation of disease severity, prevention of infection and even protection of unvaccinated population through herd immunity [8]. Thus, the availability of COVID-19 vaccine(s) will drastically change the course of the pandemic. As of 9 September 2020, 35 vaccine candidates are in clinical evaluation and 145 vaccine candidates are in preclinical evaluation [13].

Despite the immense benefits that vaccination has offered since the first discovery of the smallpox vaccine by Edward Jenner till date, saving millions of lives globally and doing so at a comparatively low cost, vaccine hesitancy has always plagued this great discovery. Vaccine hesitancy is “the reluctance or refusal to vaccinate despite the availability of vaccines” [14]. It was classified as one of the top ten threats to global health by the World Health Organization in 2019 [14]. Vaccine hesitancy is a complex phenomenon, with a growing continuum between vaccine acceptance and refusal. Despite the proven effectiveness and safety of vaccines, an increasing number of individuals perceive vaccines as unsafe and unnecessary [15]. In recent times, there has been a steady decline in vaccine coverage and an increase in the occurrence of vaccine-preventable diseases. For instance, there has been a 30% rise in measles cases globally. Vaccine hesitancy is believed to contribute greatly to this [14,15].

Nigeria is the most populated country in Africa and has a convoluted history of vaccine hesitancy. Vaccination coverage in Nigeria has continuously dropped since its peak of 81.5% in the 1990s, and by 2013, only 25% of children under the age of 2 were fully vaccinated [16]. The 2003/2004 polio vaccine refusal in Nigeria had a far-reaching effect. It increased the incidence of polio by many folds in Nigeria and contributed to outbreaks of polio across three other continents [17]. Vaccine hesitancy could have a direct and wide-reaching effect on the acceptance of COVID-19 vaccine(s) by individuals in the community as it confers threat not only on the hesitant individual but on the community as a whole, as delays and refusals would make it impossible for communities to reach the threshold of vaccine uptake necessary for the conferment of herd immunity. While the focus of attention currently is on developing a vaccine to protect the population against COVID-19, stakeholders should prepare for the next challenge: vaccine adoption (access and acceptance) among the public. Our study aimed to understand the perception of social media users in Nigeria regarding hypothetical COVID-19 vaccine.

## Methods

**Study design and sampling technique:** we conducted a web-based cross-sectional survey among males and females who use social media in Nigeria. We used a non-probability convenient sampling technique to recruit the respondents, who were required to fill the questionnaire within the first-one-month period stipulated for the study as used in previous studies [18,19]. The inclusion criteria were being a social media user, a Nigerian and having access to internet connection to fill out the online questionnaire. We excluded individuals who do not consent to participate in the study and are younger than 15 years of age [18].

**Sample size estimation:** the survey was online-based and totally depended on the voluntary participation of all the eligible respondents, so no prior sample size was calculated, yet a total of 517 respondents took part in the study. This is further justified by the non-probability convenient sampling used in the study as well as the need for caution in findings generalization as stated in the study limitation.

**Study instrument and administration:** the researchers developed the study questionnaire in several stages of drafts and reviews and with contributions from survey research experts. We conducted a pretest among ten respondents from the six geopolitical zones in Nigeria using the drafted questionnaire. The pretest was undertaken to examine readability, comprehensibility, and face validity. The final questionnaire comprises sections on the demographic characteristics of the respondents (independent variables), the intention to accept a COVID-19 vaccine was measured using a one-item question (If a vaccine against COVID-19 becomes available, would you take it? - with a Yes or No option), another question on the reasons for not intending to take the vaccine and a question on whether they have reservations towards vaccination - with a Yes or No option (outcome variables). According to our study, having reservation towards vaccination is referred to as having doubt on the benefits vaccination other than the COVID-19 vaccination can reap for public health while willingness to take COVID-19 vaccine is referred to as the acceptance of COVID-19 vaccine and the readiness to be vaccinated when it is available.

The final questionnaire was entered into an online survey system, and a link to the electronic questionnaire was shared using social media platforms, specifically WhatsApp, LinkedIn, Twitter, and Facebook, as used in a previous survey [18]. In addition to this, we urged our social media networks to share the electronic questionnaire with their networks. This was done to facilitate the achievement of more respondents. The investigators´ decision to collect the data using online survey was predicated by the need to maintain physical distance. Data collection was conducted in August 2020.

**Data analysis:** data were coded and abstracted into the Microsoft Excel spreadsheet and loaded into the STATA 14 software for final analysis. Simple descriptive analysis, including frequencies and percentages, was computed for demographic characteristics, and the reasons for non-acceptance of the COVID-19 vaccine, intention to take vaccine and reservations toward vaccination were presented with bar charts. A Chi-square test was carried out to determine the significant level of association and the relationship between the independent variables and outcome variables with statistical significance defined at P < 0.05.

**Ethical consideration:** no formal ethical approval was collected, however the researchers adhered strictly to all the ethical issues involved in conducting studies involving human participants such as informed consent which was obtained electronically. Confidentiality and anonymity were also ensured by not putting names or attaching any identifiable codes to the online questionnaires, and the rights of the participants to withdraw anytime from the study were also clearly stated in the online survey, if they were not comfortable in answering the questions.

## Results

**Socio-demographic characteristics of the respondents:** Table 1 showed that more than half of the respondents were male, 294 (56.9%). The majority 478 (92.5%) of the respondents were between 16 to 30 years of age, while most of these respondents were students 302 (58.4%). The geographical location of the respondents showed that many of the respondents were from South-West 298 (57.6%).

**Table 1:** socio-demographic distribution of respondents

Variable	N=517	
**Sex**	**Frequency**	**Percentage (%)**
Female	223	43.1
Male	294	56.9
**Age group (years)**		
16-30	478	92.5
31-45	29	5.6
46-60	6	1.2
61+	4	0.7
**Employment**		
Employed	179	34.6
Student	302	58.4
Unemployed	36	7.0
**Geo-political Zone**		
North-Central	77	14.9
North-East	6	1.2
North-West	12	2.3
South-South	69	13.4
South-East	55	10.6
South-West	298	57.6
**Education Level**		
Graduate	171	33.1
Postgraduate	52	10.0
Secondary	15	2.9
Undergraduate	279	54.0

**Figure 1:**
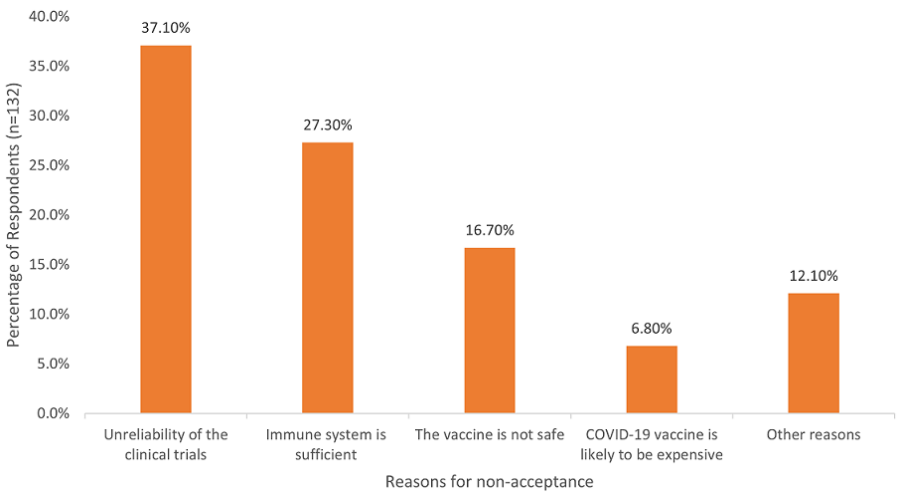
reasons for non-acceptance of the COVID-19 vaccine by our respondents (n=132)

**Percentage distribution of respondents´ reservation toward vaccination, acceptance of COVID-19 vaccine and reasons for non-acceptance of COVID-19 vaccine:** the results showed that 385 (74.5%) were willing to receive COVID-19 vaccine while 132 (25.5%) of our respondents were not willing to take the vaccine. 124 (24.0%) had reservations toward vaccination while 393 (76.0%) respondents did not have any reservations toward vaccination. The major reason for non-acceptance of the COVID-19 vaccine among our respondents is unreliability of the clinical trials 49 (37.1%), followed by the belief that their immune system is enough to combat the virus 36 (27.3%) as shown in Figure 1.

**Association between selected socio-demographic variables, having reservations toward vaccination and COVID-19 vaccine acceptance:** our result revealed having reservations toward vaccination *(χ^2^=0.10 P-value=0.76)* and COVID-19 vaccine acceptance *(χ^2^=1.53 P-value=0.22)* are not statistically associated with the sex of respondents. Both COVID-19 vaccine acceptance *(χ^2^=24.33 P-value=0.00)* and reservation towards vaccination *(χ^2^= 19.04 P-value=0.00)* are significantly associated with the age group. Only COVID-19 vaccine acceptance *(χ^2^=13.78 P-value=0.02)* is significantly associated to the respondents´ geopolitical zone. Having reservation towards vaccination *(χ^2^=12.01 P-value=0.00)* is also significantly associated to the respondents´ education level (Table 2).

**Table 2:** association between selected socio-demographic variables, reservation toward vaccination and COVID-19 vaccine acceptance

Variable N=517	Do you have any reservations toward vaccination? (Reservations toward vaccination)			Will you take COVID-19 vaccine? (COVID-19 vaccine acceptance)	
**Sex**	No n (%)		Yes n (%)	No n (%)	Yes n (%)
Female (223)	171 (76.7)		52 (23.3)	63 (28.3)	160 (71.7)
Male (294)	222 (75.5)		72 (24.5)	69 (23.5)	225 (76.5)
	**χ2**=0.10 P-value=0.76			**χ2**=1.53 P-value=0.22	
**Age group (years)**					
16-30 (478)	369 (77.2)		109 (22.8)	113 (23.6)	365 (76.4)
31-45 (29)	22 (75.9)		7 (24.1)	10 (34.5)	19 (65.5)
46-60 (6)	2 (33.3)		4 (66.7)	5 (83.3)	1 (16.7)
61+ (4)	0 (0.0)		4 (100.0)	4 (100.0)	0 (0.0)
	**χ2**= 19.04 P-value=0.00*			**χ2**=24.33 P-value=0.00*	
**Employment**					
Employed (179)	125 (69.8)		54 (30.2)	46 (25.7)	133 (74.3)
Student (302)	243 (80.5)		59 (19.5)	74 (24.5)	228 (75.5)
Unemployed (36)	25 (69.4)		11 (30.6)	12 (33.3)	24 (66.7)
	**χ2**=7.88 P-value=0.02*			**χ2**=1.32 P-value=0.52	
**Geo-political**					
North-Central (77)	55 (71.4)		22 (28.6)	20 (26.0)	57 (74.0)
North-East (6)	6 (100.0)		0 (0.0)	2 (33.3)	4 (66.7)
North-West (12)	7 (58.3)		5 (41.7)	5 (41.7)	7 (58.3)
South-South (69)	51 (73.9)		18 (26.1)	14 (20.3)	55 (79.7)
South-East (55)	41 (74.5)		14 (25.5)	24 (43.6)	31 (56.4)
South-West (298)	233 (78.2)		65 (21.8)	67 (22.5)	231 (77.5)
	**χ2**=5.84 P-value=0.32			**χ2**=13.78 P-value=0.02*	
**Education Level**					
Graduate (171)	129 (75.4)		42 (24.6)	39 (22.8)	132 (77.2)
Postgraduate (52)	30 (57.7)		22 (42.3)	20 (38.5)	32 (61.5)
Secondary (15)	11 (73.3)		4 (26.7)	6 (40.0)	9 (60.0)
Undergraduate (279)	223 (80.0)		56 (20.0)	67 (24.0)	212 (76.0)
	**χ2**=12.01 P-value=0.00*			**χ2**=7.22 P-value=0.06	
**Total**	**393 (76.0)**	**124 (24.0)**		**132 (25.5)**	**385 (74.5)**

* significant at P<0.05

## Discussion

This study is the first in Nigeria to assess public perception towards a hypothetical COVID-19 vaccine. It has been documented that the public perception of vaccine governs the effectiveness of vaccination programs [20]. There is no specific antiviral treatment for COVID-19, and many vaccine candidates are currently under clinical trials [1]. Vaccination, one of the greatest advances in medicine, is one of the most effective tools for reducing the burden of infectious diseases. Over the last two decades, worldwide, vaccination programs for polio, whooping cough, diphtheria, and measles have significantly reduced the prevalence of these diseases [9]. Despite the benefits vaccination reap for public health, this fundamental effort for disease control still faces major obstacles globally, and Nigeria is not an exception [21]. It has been noted that one of the major obstacles to vaccine acceptance is the public perception of the relative risks and benefits of vaccination [22].

Findings from our study revealed that there is no significant association between COVID-19 vaccine acceptance and sex. This contrasts with a multi-national survey in Europe, where there was a significant association between the two variables and a significantly higher proportion of males were willing to get vaccinated [23]. Even though there is no significant difference in the willingness to take the COVID-19 vaccine and sex, males are slightly willing to take the COVID-19 vaccine than females. Our results also revealed a significant association between age group and COVID-19 vaccine acceptance, with respondents age 16 to 30 more willing to take the COVID-19 vaccine. One might argue that the group who currently disagreed with taking the COVID-19 vaccine may be the most relevant. However, these sets of people can easily be persuaded to get vaccinated to achieve herd immunity; if they are informed of the benefits vaccination can reap towards containing the pandemic.

Regarding the COVID-19 vaccine acceptance, about 74% of our respondents agreed to take the COVID-19 vaccine when it is available. This is higher than what was reported in South Africa (64%), Russia (54%), Poland (56%), Hungary (56%), and France (59%), according to a recent World Economic Forum´s Ipsos survey of nearly 20,000 adults on whether they will take COVID-19 vaccine or not when it is available [24]. However, the willingness to take the COVID-19 vaccine was higher in China (97%), Brazil (88%), Australia (88%), and India (87%) [24] compared to our study. A study in Indonesia also reported that for a 95% effective COVID-19 vaccine, 93.3% would take it [25]. Another study in Malaysia revealed that 94.3% of the respondents would take the COVID-19 vaccine [26]. The acceptance level of COVID-19 vaccine among our respondents is higher than a study in Democratic Republic of Congo, where 28% would take the vaccine should it be available [27]. Data from the survey conducted by the Africa Centres for Disease Control and Prevention (Africa CDC), in partnership with the London School of Hygiene & Tropical Medicine also shows major variations in willingness across countries and across the five regions in the continent, from 94% and 93%, respectively, in Ethiopia and Niger to 65% and 59%, respectively, in Senegal and the Democratic Republic of Congo [28].

Finding from our study also revealed that geographical location and acceptance of the COVID-19 vaccine are significantly associated. This is evident in that respondents from the Southern part of the country are likely to take the COVID-19 vaccine compared to the Northern part of the country. This is not surprising because previous studies have reported high levels of vaccine refusal in northern part of Nigeria [29,30]. For instance, in 2003, five northern Nigerian States boycotted the oral polio vaccine due to fears that it was unsafe [17]. Other plausible reasons for the refusal maybe due to low level of education resulting into poor health literacy, distrust in vaccine, distrust in government due to conflicts and insecurities, possible influence of cultural and religious beliefs among others. However, these findings do not necessarily imply that refusal would be higher in Northern Nigeria; it thus strengthens the need to reassure the public of the safety of the COVID-19 vaccine irrespective of their geographical location. The acceptance level of the COVID-19 vaccine, according to our study, is lower than that of the hypothetical Ebola vaccine (80%) [31] and malaria vaccine (96%) [32] in previous studies conducted in Nigeria.

Furthermore, about 25% of our respondents disagreed with taking the COVID-19 vaccine when it is available. This is higher than what was reported in China (3%), Brazil (12%), Australia (12%), India (13%), Malaysia (15%), Great Britain (15%), Saudi Arabia (16%), South Korea (16%), Peru (21%), and Canada (24%) according to a recent World Economic Forum´s Ipsos survey of nearly 20,000 adults on whether they will take COVID-19 vaccine or not when it is available [24]. However, the unwillingness to take the COVID-19 vaccine was higher in Russia (47%), Poland (45%), Hungary (44%), France (41%), South Africa (36%), Sweden (33%), United States (33%), Germany (33%) and Italy (33%) [24] compared to our study. A study by the Africa CDC also revealed that 5%, 7% and 35% of the respondents would not take COVID-19 vaccine in Ethiopia, Niger, and Senegal respectively [28].

Findings from our study also revealed that the top three reasons for non-acceptance of the COVID-19 vaccine are the unreliability of clinical trials, the immune system is enough to combat COVID-19, and that the COVID-19 vaccine is not safe. This is similar to what was reported by the Ipsos survey, where the top 3 reasons were “worry about side effects”, “doubt about the vaccine effectiveness”, and “perception of not being enough at risk from COVID-19” [24]. Also, in a survey by Africa CDC across 15 African countries, the reasons for not accepting COVID-19 vaccines depended mostly on trust in vaccines as well as perceptions of its importance, safety, and efficacy [28]. A multi-national study in Europe also revealed that more than half said they were concerned about the potential side effects of the vaccine [23]. This is not surprising in that all these findings have been found in literature as some of the reasons for vaccine hesitancy [33,34]. It has also been documented in the literature that vaccine adoption is a sum of vaccine access and acceptance [35].

Findings from this study revealed 25% of our respondent will not accept the COVID-19 vaccine. Thus, there is a possible implication of influencing others with their perception of the COVID-19 vaccine leading to more people refusing the vaccine. This could lead to widespread refusal of COVID-19 vaccine due to eroded public trust with the negative information regarding the COVID-19 vaccine. The estimated reproductive number of COVID-19 for Nigeria has been determined to be 2.63 [36]. Going by the formula Q = 1 - 1/R, where Q is the herd immunity threshold and R is the reproductive number [37], an estimated 62% or more of the Nigerian population must be vaccinated to achieve herd immunity. For Nigeria to achieve this, national health authorities need to devise means to ensure the public trust is earned and all communities, including the marginalized populations, must be engaged properly to ensure an increase in vaccine acceptance. In addition, all information regarding the vaccine must be made public, quality control and assurance must also be the priority of the health authorities. This further heightened the need to strengthen and invest in COVID-19 vaccine communication and community engagement [38,39].

Interestingly, one of the reasons for non-acceptance of COVID-19 vaccine is the vaccine´s perceived high cost according to our findings. Access is also an important factor to be considered as regards the COVID-19 vaccine [40,41], and it is essential to translate the willingness to be vaccinated into actual vaccination decisions when the vaccine becomes available. Even though our study did not assess the willingness to pay for the COVID-19 vaccine, equitable access to vaccination is much-needed to quickly achieve herd immunity.

**Limitations:** our study is not without its limitations. Generalization of the survey results should be avoided because the pattern of questionnaire distribution may influence the outcome. We used the social media platform, so it may omit older adults, people from lower socioeconomic classes, certain geographical locations, lower educational attainment, and those who were illiterates as well as people who did not have access to the internet. Besides, response/social bias is also a possibility which is not uncommon in self-administered questionnaire research. Finally, acceptance was assessed using a potential (hypothetical) vaccine, which may differ from the respondents' revealed preferences when the vaccine becomes available. Thus, future study may need to put all these gaps into consideration to ensure a far-reaching conclusion in this regard.

## Conclusion

Even though most of our respondents are willing to take the COVID-19 vaccine, our findings reiterate the need to reassure the public the benefits an effective and safe COVID-19 vaccine can reap for pandemic response. Otherwise, there is a risk of a reversal of hard-won achievement of building public trust in Nigeria´s vaccination programme, potentially compromising reaching community immunity. Public trust is important in promoting public health and plays an essential role in the public's compliance with vaccination programs and other health interventions. However, if the public trust is eroded, false information can spread, leading to the rejection of health interventions with a major threat to public health. It is also important that Nigerian government should heighten their investment in effective and clear vaccine communication and community engagement. Besides, national health authorities, stakeholders and policymakers in Nigeria need to ensure that access to the COVID-19 vaccine is equitable when it becomes available.

### What is known about this topic

- COVID-19 pandemic is a global public health threat facing mankind;
- The availability of COVID-19 vaccine will reap enormous benefits to effectively contain the pandemic;
- There are presently many vaccine candidates under development and some of these vaccine candidates are in Phase III clinical trials.

### What this study adds

- Majority of our respondents are willing to take COVID-19 vaccine when it becomes available;
- The top three reasons for non-acceptance of the COVID-19 vaccine among our respondents include: unreliability of clinical trials, the immune system is enough to combat COVID-19, and that the COVID-19 vaccine is not safe;
- Our findings reiterate the need to reassure the public the benefits an effective and safe COVID-19 vaccine can reap for public health. Earning public trust is essential for optimal COVID-19 vaccine acceptance.
